# Design of a Honey Badger Optimization Algorithm with a Deep Transfer Learning-Based Osteosarcoma Classification Model

**DOI:** 10.3390/cancers14246066

**Published:** 2022-12-09

**Authors:** Thavavel Vaiyapuri, Akshya Jothi, Kanagaraj Narayanasamy, Kartheeban Kamatchi, Seifedine Kadry, Jungeun Kim

**Affiliations:** 1 Department of Computer Science, College of Computer Engineering and Sciences, Prince Sattam Bin Abdulaziz University, Al Kharj 16278, Saudi Arabia; 2 Department of Computational Intelligence, SRM Institute of Science and Technology, Kancheepuram 603203, India; 3 Department of Computer Science, Karpagam Academy of Higher Education, Coimbatore 641021, India; 4 Department of Computer Science and Engineering, Kalasalingam Academy of Research and Education, Krishnankoil 626126, India; 5 Department of Applied Data Science, Noroff University College, 4612 Kristiansand, Norway; 6 Artificial Intelligence Research Center (AIRC), Ajman University, Ajman 346, United Arab Emirates; 7 Department of Electrical and Computer Engineering, Lebanese American University, Byblos P.O. Box 13-5053, Lebanon; 8 Department of Software, Kongju National University, Cheonan 31080, Republic of Korea

**Keywords:** deep learning, metaheuristics, honey badger algorithm, osteosarcoma classification, medical imaging

## Abstract

**Simple Summary:**

Osteosarcoma is one of the aggressive bone tumors with numerous histologic patterns. Histopathological inspection is a crucial criterion in the medical diagnosis of osteosarcoma. Due to the advancement of computing power and hardware technology, pathological image analysis system based on artificial intelligence were more commonly used. Manual examination of the histopathological images is a difficult and laborious task. The lack of labeling data makes the system difficult to build and costly. Therefore, this study aims to develop an automated computer-aided diagnosis model for osteosarcoma classification. The proposed model uses deep learning, hyperparameter optimizer, and fuzzy logic for the classification process.

**Abstract:**

Osteosarcoma is one of the aggressive bone tumors with numerous histologic patterns. Histopathological inspection is a crucial criterion in the medical diagnosis of Osteosarcoma. Due to the advancement of computing power and hardware technology, pathological image analysis system based on artificial intelligence (AI) were more commonly used. But classifying many intricate pathology images by hand will be challenging for pathologists. The lack of labeling data makes the system difficult to build and costly. This article designs a Honey Badger Optimization with Deep Learning based Automated Osteosarcoma Classification (HBODL-AOC) model. The HBODL-AOC technique’s goal is to identify osteosarcoma’s existence using medical images. In the presented HBODL-AOC technique, image preprocessing is initially performed by contrast enhancement technique. For feature extraction, the HBODL-AOC technique employs a deep convolutional neural network-based Mobile networks (MobileNet) model with an Adam optimizer for hyperparameter tuning. Finally, the adaptive neuro-fuzzy inference system (ANFIS) approach is implemented for the HBO (Honey Badger Optimization) algorithm can tune osteosarcoma classification and the membership function (MF). To demonstrate the enhanced classification performance of the HBODL-AOC approach, a sequence of simulations was performed. The extensive simulation analysis portrayed the improved performance of the HBODL-AOC technique over existing DL models.

## 1. Introduction

Osteosarcoma is a cancer that originates from the bone and grows quickly to form cancerous bone-like tissue and is an orthopedics disease. Commonly, osteosarcoma arises at the upper end of the humerus, the lower end of the femur, and the upper end of the tibia, particularly around the knee joint [1]. Osteosarcoma mostly occurs in children and adolescents, and its indications consist of fever, mild bone pain, and redness at the cancer site. Constant pain caused by osteogenic sarcoma affects the movement of the patient, and therefore it is a significant cancer that extremely affects labor efficiency and threatens life [2]. Thus, initial treatment and diagnosis have specific importance. Current diagnostic approaches, which include computer tomography (CT), magnetic resonance imaging (MRI), positron emission tomography (PET), and ultrasound, play a vital role in cancer identification [3]. But if such methods cannot earn a precise judgment, clinicians desire to derive tissue samples from cancer for further investigation. Concretely, the derived examples are transformed into smears or slides, and after stained to display some details regarding cells, which is generally time-consuming and leads to great pain in patients [4]. Hence, the growth of automated recognition technology for osteosarcoma has a higher value. Owing to the rise of patient-specific treatment options and cancer incidence, medication and the diagnosis of cancer have become very complex [5]. Diagnosticians should spend a long time analyzing more slides. Identifying the nuances of histologic images was tough. Misdiagnosis frequently happens because of extensive work that declines the precision of diagnosis [6]. The osteoblasts’ morphologies have minute variance in distinguished cells, which makes the image hardly distinguishable. Similarly, a biopsy will be a dynamic and time taking step for determining the existence of malignant tissue [7]. In the meantime, to automatically identify malignancies.

Computer-Aided Detection (CAD) technology renders a solution for radiotherapists. Extraction of features will be the next step in the automatic identification mechanism, and it is executed by deep learning (DL) or manually [8]. Handcrafted (HC) features were picture-specific properties decided by hand depending on targeted features of spaces, and such techniques were broadly used for extraction. The authors made widespread usage of HC characteristics since they were easy to derive, specifically in modest databases. The features are determined with professionals’ help in the appropriate sector [9]. Owing to their difficulty, such characteristics are problematic in deciding if they are associated with complicated images. In this case, DL methods were employed as a feature extraction algorithm. Because of recent advancements in the area of processing, such as the advent of quick and compact processors, the DL model has received significant attention in recent years, enabling experts to quickly and easily train deeper networks [10].

This article designs a Honey Badger Optimization with Deep Learning based Automated Osteosarcoma Classification (HBODL-AOC) model. The goal of the presented HBODL-AOC technique is to identify the existence of osteosarcoma using medical images. In the presented HBODL-AOC technique, image preprocessing is initially performed by contrast enhancement technique. For feature extraction, the HBODL-AOC technique employs deep convolutional neural network-based mobile networks (MobileNet) model with an Adam optimizer for hyperparameter tuning. Finally, the adaptive neuro-fuzzy inference system (ANFIS) approach is implemented for the HBO algorithm can tune osteosarcoma classification and the membership function (MF). To demonstrate the enhanced classification performance of the HBODL-AOC technique, a sequence of simulations was performed. In short, the key contributions of the paper are given as follows.

- An intelligent HBODL-AOC technique comprising pre-processing, MobileNet feature extraction, an Adam optimizer, ANFIS classifier, and HBO-based parameter tuning is presented. To the best of our knowledge, the HBODL-AOC model has never been presented in the literature;
- Employ the MobileNet model with an Adam optimizer to generate a useful set of feature vectors;
- Present an ANFIS model for osteosarcoma classification with HBO algorithm as a parameter optimization technique. Parameter optimization of the ANFIS model using the HBO algorithm using cross-validation helps to boost the predictive outcome of the HBODL-AOC model for unseen data.

## 2. Related Works

Pan et al. [11] propose a classical transformer image classification architecture with the integration of feature cross fusion learning (FCFL) and noise reduction convolutional autoencoder (NRCA) for classifying osteosarcoma histological images. NRCA could denoise histological images of osteosarcoma, which leads to more pure images for osteosarcoma segmentation. Furthermore, the research workers presented feature cross fusion learning that incorporates two scale image patches to considerably explore their interaction with other classification tokens. Ling et al. [12] developed an intellectually assisted diagnosis technique for osteosarcoma that could decrease the workload of clinicians in identifying osteosarcoma from three features. Firstly, the research workers constructed a classification-image enhancement method comprising resnet18 and DeepUPE to enhance image clarity and eliminate redundant images that could facilitate doctor observation. Next, the research workers empirically compare the performances of hybrid, serial, and parallel fusion convolution and transformer and present a double U-shaped visual transformer with convolution (DUconViT) for automated classification of osteosarcoma to help doctor diagnoses.

In [13], a robust detection technique has been introduced based on Fractional-Harris Hawks Optimization (F-HHO) related generative adversarial network (GAN) to detect osteosarcoma at an earlier phase. Now, the presented method was intended by the incorporation of HHO and Fractional Calculus, correspondingly. GAN is utilized for performing osteosarcoma recognition on the basis of features derived from the images by using the cell classification method. In [14], proposed a new method for the calculation of tumor stages and grade in long bones relevant to X-ray image analysis. Usually, cancer-affected bone images appear with the variation in bone texture in the affected area. In this work, the author extracts specific feature from bone X-ray image and utilize a support vector machine (SVM) to discriminate between cancerous and healthy bones. Abdelaal and Tobely [15] developed particle swarm-optimized extreme learning neural networks for efficiently forecasting bone cancers. At first, an X-ray image was collected from the oral cancer dataset that should be inspected for noise to remove by means of a non-local median filter. The extracted feature was categorized as a particle swarm optimization-based Extreme Learning Neural Networks Classifier.

Wu et al. [16] developed a boundary-aware grid contextual attention net (BA-GCA Net) to resolve the problems of inadequate performance in osteosarcoma MRI image classification. Firstly, a grid contextual attention (GCA) was intended for capturing texture details of the tumor region. Next, the spatial transformer block (STB) and statistical texture learning block (STLB) are incorporated with the networks to enhance the capability for extracting statistical texture features and locating tumor regions. The author [17] developed an automated bone cancer diagnosis technique to predict cancer at an earlier stage. Firstly, the bone image was gathered from the patients, and noise in the image was removed by means of a median filter. Afterward removing the noise, the affected tumor region can be diagnosed by employing the intuitionistic fuzzy rank correlation. Distinct statistical features were extracted from the diagnosed intuitionistic fuzzy-based clustered images. The obtained feature was processed with the help of a deep neural network (DNN) layer that effectively investigates every feature using the Levenberg–Marquardt learning model.

In spite of the several DL models that existed in the earlier studies, it is still needed to enhance the osteosarcoma classification performance. Due to the incessant deepening of the model, the number of parameters of DL models gets increased, and it leads to model overfitting. Besides, various hyperparameters have a substantial influence on the performance of the CNN model. Principally, hyperparameters such as epoch count, batch size, and learning rate selection are essential to attain effectual outcomes. Since the trial and error method for hyperparameter tuning is a tiresome and inaccurate process, hyperparameter optimizers can be applied. On the other hand, the choice and shape of MFs affect the performance of the fuzzy system irrespective of the significance. Therefore, in this work, an Adam optimizer and HBO algorithm are applied for the parameter optimization of the MobileNet and ANFIS models, respectively.

## 3. The Proposed Model

**Figure 1:**
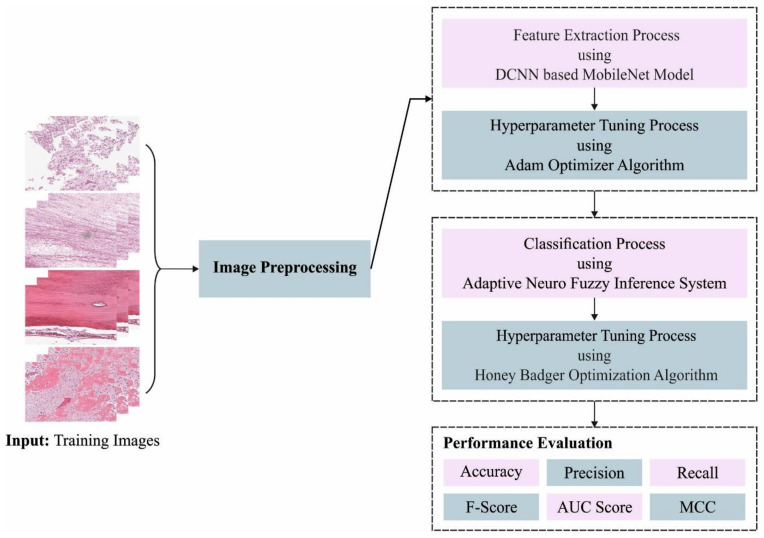
Working process of HBODL-AOC algorithm. DCNN: deep convolutional neural network; MCC: Mathew Correlation Coefficient.

In this article, we have introduced an Automated Osteosarcoma Classification model named the HBODL-AOC model. The goal of the presented HBODL-AOC technique is to identify the existence of osteosarcoma using medical images. In the presented HBODL-AOC technique, different sub-processes are involved, namely contrast enhancement, deep convolutional neural network (DCNN) based MobileNet feature extraction, Adam optimizer, ANFIS classification, and HBO-based parameter tuning. Figure 1 depicts the working procedure of the HBODL-AOC approach.

### 3.1. Image Pre-Processing

In the presented HBODL-AOC technique, image pre-processing is initially performed by contrast enhancement technique. Contrast enhancement approaches have progressed in the past few decades to address the requirements of its objectives. There were 2 key goals in improving an image’s contrast. One is facilitating or increasing the efficiency of subsequent tasks (for example, image segmentation, image analysis, and object detection), and another one is improving appearance for visual interpretation. Many contrast enhancement methods depend on histogram modifications, which are implemented locally or globally. The method that overcomes the limitations of global techniques by enhancing local contrast is called the Contrast Limited Adaptive Histogram Equalization (CLAHE) technique [18]. CLAHE was a variation of Adaptive histogram equalization (AHE) that thwarts contrast over-amplification. CLAHE operates on smaller areas of an image known as tiles instead of the complete image. The surrounding tiles can be combined through bilinear interpolation to remove the false boundaries. This method is employed for enhancing image contrast.

### 3.2. Feature Extraction

At this stage, the MobileNet model is applied for feature extraction. CNN is mostly collected from fully connected (FC) input, pooling, output, and convolution layers [18]. As compared to the typical neural network, it features local connection, down-sampling, and weighted sharing. It could efficiently decrease the network parameter, avoid over-fitting, and enhance the efficacy of removing local features. The convolutional layer was a basic element of the convolutional neural network (CNN), and the local extracting feature was recognized by linking the input of all the neurons to the local sensing area of the preceding layer. The convolutional function is classified as convolution and activation, and the computation procedure is demonstrated as:

(1) $$T=f_{k}\left(\overset{r}{\underset{x,y,z=1}{\sum }}C_{x,y,z}w_{x,y,z}^{s}+b^{s}\right)$$

where C and T signify the input and resultant of the convolutional layer correspondingly; r and s represent the serial number of convolutional kernels, and the channel counts correspondingly; w and b denote the weight as well as the bias of the convolutional kernel; $f_{k}$ implies the activation function of $k^{th}$ layers; and x, y, and z represent the dimensional of input datasets.

During the activation function, non-linear function like rectified linear unit (ReLU), Sigmoid, Leaky ReLU, and Tanh is implemented for mapping the input later linear transformation for enhancing the non-linear expression capability of networks. Especially, ReLU removes the gradient vanishing outcome of the sigmoid purpose, and the gradient computation speed was very quick; thus, it can be extremely utilized. Thus, the ReLU was executed to the convolutional layer under this work. The pooling layer has a feature mapping layer that decreases the resultant dimensional of the convolutional layer for realizing the down-sampling of local data and efficiently avoiding over-fitting. Overlapping pooling, max pooling, and average pooling can be general pooling approaches. During this case, max pooling was implemented for expressing local features, and several convolutional and pooling layers can be utilized for realizing extracting features.

During the fully connected (FC) layer, all the neurons are FC to every neuron from the front layer, and the predictive value was computed by weight summation of inter-layer weighted co-efficient. To regression procedures, the non-linear activation functions like Sigmoid, ReLU, and Tanh could not be appropriate to the final FC layer. While it maps the outcome in the range of (0, ∞), $\left(-1,1\right)$, and $\left(0,1\right)$, correspondingly. So, for improving the expression capability of the method, ReLU and linear activation functions can be executed to FC and output layers correspondingly.

MobileNetV2 is a mobile-enhanced FC network and relies on the inverted residual architecture, which has a bottleneck level interconnected to residual connection [19]. A first FC layer with 32 filters is employed in the MobileNetV2 that can be followed by 19 residual bottleneck layers. Six stages were followed in the model progression, which generates the amplification image generator, fundamental method with MobileNetV2, training the model, building up the model, storing model for forthcoming approximation, and process adding model parameters. A loss of 0.25 assured a random exclusion of 25% of the weight during training. This method significantly reduces overfitting. The major aim is to retain from utilizing too many weights models and from gaining a widespread knowledge of the input. For these datasets, a batch size of 32 images was exploited. Accordingly, 32 images were learned in one cycle. Commonly, the model grows large once the batch size is enhanced. However, this reduces the module’s ability to classify uncommon classes. Over an extensive size of the model, MobileNetV2 enhances efficacy. The MobileNetV2 is encompassed of n times as numerous recurrent layers. In this work, depthwise separable convolutions are used, which consist of depthwise and pointwise convolutions after one another.

The hyperparameter tuning process is performed by the Adam optimizer. It is a kind of typical stochastic gradient descent (SGD) method for upgrading network weighted in trained data [20]. It can be utilized for performing optimization and is the most optimum optimizer at the moment. Adam proceeds in adagrad, and it can be a further adaptable manner. Adagrad and momentum combined are called Adam.

Parameters $w^{\left(t\right)}$ and $L^{\left(t\right)}$, whereas index t signifies the present trained iteration, Parameter upgrade in Adam can provide as:

(2) $$m_{w}^{\left(t+1\right)}\leftarrow \beta_{1}m_{w}^{\left(t\right)}+\left(1-\beta_{1}\right)\nabla_{w}L^{\left(t\right)}$$

(3) $$v_{w}^{\left(t+1\right)}\leftarrow \beta_{2}v_{w}^{\left(t\right)}+\left(1-\beta_{2}\right){\left(\nabla_{w}L^{\left(t\right)}\right)}^{2}$$

(4) $${\hat{m}}_{w}=\frac{m_{w}^{\left(t+1\right)}}{1-{\left(\beta_{1}\right)}^{\left(t+1\right)}}$$

(5) $$V_{w}=\frac{v_{w}^{\left(t+1\right)}}{1-{\left(\beta_{2}\right)}^{\left(t+1\right)}}$$

(6) $$w^{t+1}\leftarrow w^{t}-\eta \frac{\hat{m}}{\sqrt{{\hat{v}}_{w}}+\in }$$

In Equations (2) and (3), $\beta_{1}$ and $\beta_{2}$ denotes the gradient forgetting features and the second moment of gradients. In Equation (6), ∈ implies the smaller scalar utilized for preventing division by 0.

### 3.3. Osteosarcoma Classification Using Optimal ANFIS Model

For the identification and classification of osteosarcoma, the ANFIS model is exploited. Soft computing techniques like neural networks and fuzzy set concepts are instances of instruments that might be exploited for establishing smart systems [21]. This theory provides a new methodology to resolve the problems that probability theory was incapable of shedding light on. Furthermore, knowledge given by humans was essential for these systems. The fuzzy rule is frequently involved in fuzzy deduction architecture, the most common type of fuzzy examination and fuzzy structure. Mostly, rules might be seen as follows: They comprise fuzzy recommendations and phonetic factors.

(7) $$If<Premise\;Proposition\;\left(p\right)>Then<Consequent\;Proposition\;\left(q\right)>$$

**Figure 2:**
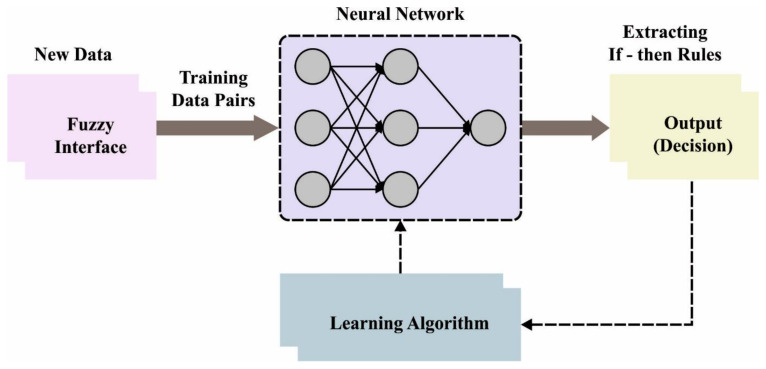
Architecture of ANFIS.

Sometimes if the rules are imposed by the regulator in the FIS, but in the ANFIS, such rules establish appropriate conditions. Once the rule cannot be followed for some reason, it should be eliminated. Likewise, the neural network accomplishes its optimum state. Note that the initial stage is represented as training, and the method displays an ideal system with the minimum error that can be remotely possible. Figure 2 showcases the framework of ANFIS.

The aim is to enhance the performance while concurrently decreasing the mistake rate and describing related error indices and functions. Fuzzy if-then rules with one output and two inputs might be formulated by:

The first rule, if $x=A_{1}$ and $y=B_{1}$ then $f_{1}=p_{1}x+q_{1}y+r_{1}$.

The second rule, if $x=A_{2}$ and $y=B_{2}$ then $f_{2}=p_{2}x+q_{2}y+r_{2}$.

Where x and y denote the input; $A_{1},$ $A_{2},$ $B_{1}$, and $B_{2}$ designates the phonological labels; p and q represent the resulting factor, and f denotes the output in fuzzy.

Now x and y characterize the input or passive layer, and membership function, rule, norm, output, and last output layers characterize the first, second, third, fourth, and fifth layers, correspondingly. Finally, the HBO algorithm is used to select the MFs optimally. In HBO, the honey badger’s (HB) dynamic searching performance with digging and honey-search tactics was separate from the exploration and exploitation phases [22]. The HB desires to live apart from the self-dug tunnel and only meets others for mating. But, because of its brave approach, it can be hunted by much greater animals once it is ineffectual to flee. However, an HB climbs a tree to access bird nests and beehives for food. An HB determines its meals by searching for mouse nests and digging, or subsequently, the honeyguide bird that realizes hives then cannot attain honey. The HBO’s mathematical structure was demonstrated as:

- The proposed HBO starts with initialized of the count of HBs dependent upon the population number $\left(Ns\right)$ and the subsequent position:
  (8) $$Y_{j}=1b_{j}+r_{1}\times \left(ub_{j}-1b_{j}\right)$$
  whereas $Y_{j}$ refers to the HB position, $lb_{j}$ and $ub_{j}$ signifies the lower as well as upper limits of all the positions from the searching space, and $r_{1}$ denotes the arbitrary number betwixt zero and one.
- The intensity (Int) has stated that it will be proportional to concentrates, prey strength, and the length betwixt the $j^{th}$ HB, as well as prey. The prey moves fast if the smell strength is stronger and different. The subsequent equation was utilized for computing the determining intensity:
  (9) $$Int_{j}=r_{2}\times \frac{SS}{4\pi d_{j}^{2}}$$
  (10) $$SS={\left(Y_{j}-Y_{j+1}\right)}^{2}$$
  (11) $$d_{j}=Y_{prey}-Y_{j}$$

In which SS signifies the source strength, $r_{2}$ denotes the arbitrary number, and $d_{j}$ represents the distance betwixt $Y_{prey}$ and the $j^{th}$ badger place. The strength in Equation (10) has assumed that the squared variance betwixt the HB’s present and next position as strength is continuously positive as it refers to intensity. The density factor $\left(\varphi \right)$ was definite and upgraded the time-varying randomized controls to ensure a smooth transition from exploration to exploitation. This feature reduces with iterations for decreasing randomized with time as:

(12) $$\varphi =Cc\times \;\exp\;\left(\frac{-iter}{iter_{\;\max\;}}\right)$$

In which $iter_{\max}$ refers to the maximal iterations number, and Cc stands for constant equivalent to two.

- To enhance the get-away from the local to the optimum area, a flag $\left(Fg\right)$ was created that changes the searching directions. Therefore, agents take availing higher chances of scanning the search space rigorously. It can be defined as:
  (13) $$Fg=\left\{\begin{array}{l} 1\;if\;r_{3}\leq 0.5\\ -1\;otherwise \end{array}\right.$$
  whereas $r_{3}$ implies the arbitrary number betwixt zero and one. Afterward, the agent positions can be upgraded whereas $Y_{new}$ was upgraded based on 2 stages digging and honey stages, as follows: during the digging stage, an HB carries out activities related to cardioid shape that is simulated as:
  (14) $$Y_{new}=Y_{prey}+Fg\times \beta \times I\times Y_{prey}+Fg\times r_{4}\times \varphi \times d_{j}\;\;\;\;\;\;\;\;\;\;\;\times \left|\cos\left(2\pi r_{5}\right).\left[1-\cos\left(2\pi r_{6}\right)\right]\right|$$

In which β implies the capability of HB for obtaining food that is superior to or equivalent to 1 (default =6) and $r_{4},$ $r_{5}$, and $r_{6}$ are 3 distinct arbitrary numbers betwixt zero and one. During the honey stage, an HB monitors a honeyguide bird for reaching a beehive that can be inspired as:

(15) $$Y_{new}=Y_{prey}+Pg\times r_{7}\times \varphi \times d_{j}$$

whereas $r_{7}$ denotes the arbitrary number betwixt zero and one.

## 4. Performance Validation

**Figure 3:**
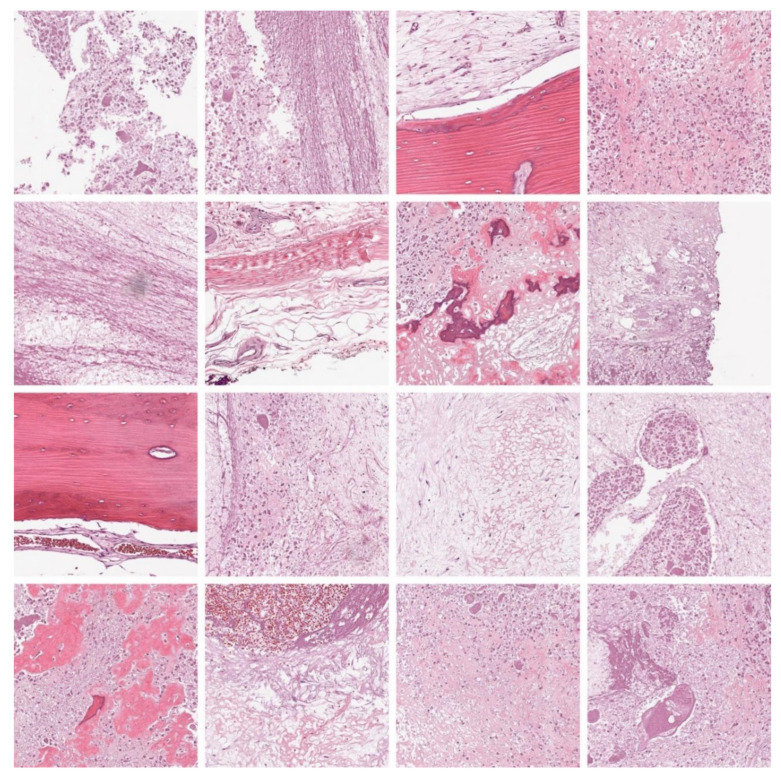
Sample images.

The proposed model is simulated using Python 3.6.5 tool. The proposed model was tested using PC i5-8600 k, GeForce 1050Ti 4 GB, 16 GB RAM, 250 GB SSD, and 1 TB HDD. The parameter settings are given as follows: learning rate: 0.01, dropout: 0.5, batch size: five, epoch count: 50, and activation: ReLU. The HBODL-AOC model is tested using a benchmark database [23] containing 1144 images under three classes. It comprises 345 images of viable tumors (VT), 263 images of non-viable tumors (NVT), and 536 images of the non-tumor (NT) class. Figure 3 illustrates the sample images.

**Figure 4:**
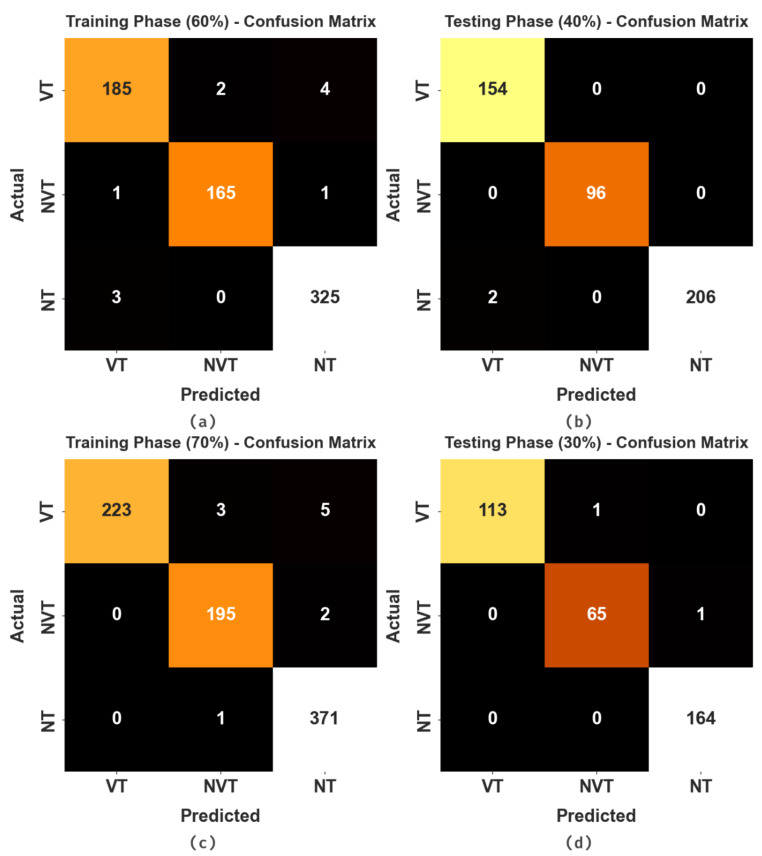
Confusion matrices of HBODL-AOC approach (**a**,**b**) 60:40 of training/testing data and (**c**,**d**) 70:30 of training/testing data.

The confusion matrices of the HBODL-AOC model on OC performance are given in Figure 4. The results implied that the HBODL-AOC model could effectively identify different class labels.

**Figure 5:**
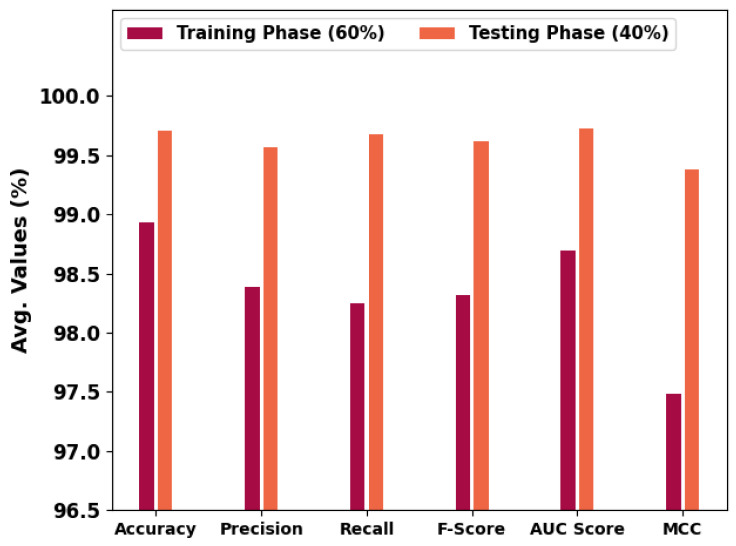
Average outcome of HBODL-AOC approach on 60:40 of TR/TS databases.

In Table 1 and Figure 5, an overall OC performance of the HBODL-AOC model under 60% of training and 40% of testing datasets is given. The results implied that the HBODL-AOC model has properly identified VT, NVT, and NT classes under both data. On 60% of the training database, the HBODL-AOC model has gained an average $accu_{y}$ of 98.93%, $prec_{n}$ of 98.39%, $reca_{l}$ of 98.25%, $F_{score}$ of 98.32%, $AUC_{score}$ of 98.69%, and Mathew Correlation Coefficient (MCC) of 97.48%. Meanwhile, on 40% of the testing database, the HBODL-AOC method has acquired an average $accu_{y}$ of 99.71%, $prec_{n}$ of 99.57%, $reca_{l}$ of 99.68%, $F_{score}$ of 99.62%, $AUC_{score}$ of 99.73%, and MCC of 99.38%.

**Figure 6:**
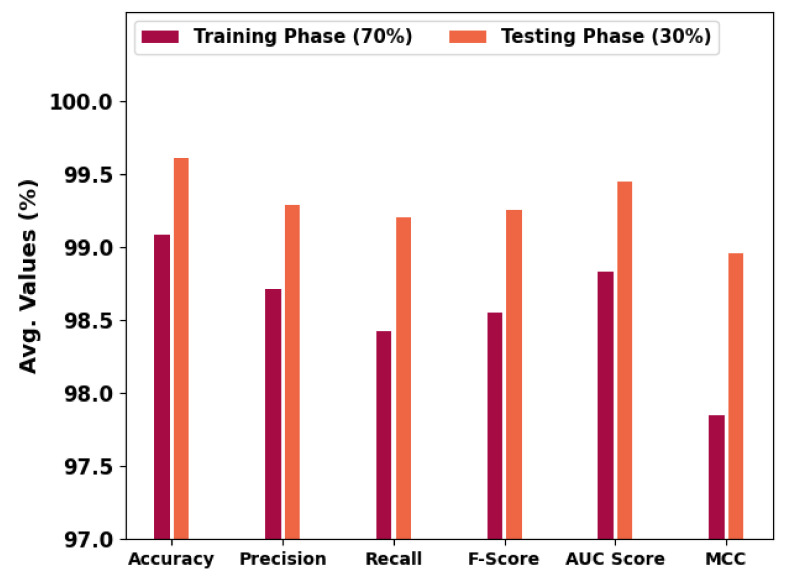
Average outcome of HBODL-AOC approach on 70:30 of TR/TS databases.

Table 2 and Figure 6 portray the overall OC performance of the HBODL-AOC model under 70% of training and 30% of the testing datasets given. The outcomes exhibited that the HBODL-AOC approach has properly identified VT, NVT, and NT classes under both data. On 70% of the training database, the HBODL-AOC technique has acquired an average $accu_{y}$ of 99.08%, $prec_{n}$ of 98.71%, $reca_{l}$ of 98.42%, $F_{score}$ of 98.55%, $AUC_{score}$ of 98.83%, and MCC of 97.85%. In the meantime, on 30% of the testing database, the HBODL-AOC methodology has obtained an average $accu_{y}$ of 99.61%, $prec_{n}$ of 99.29%, $reca_{l}$ of 99.20%, $F_{score}$ of 99.25%, $AUC_{score}$ of 99.45%, and MCC of 98.96%.

**Figure 7:**
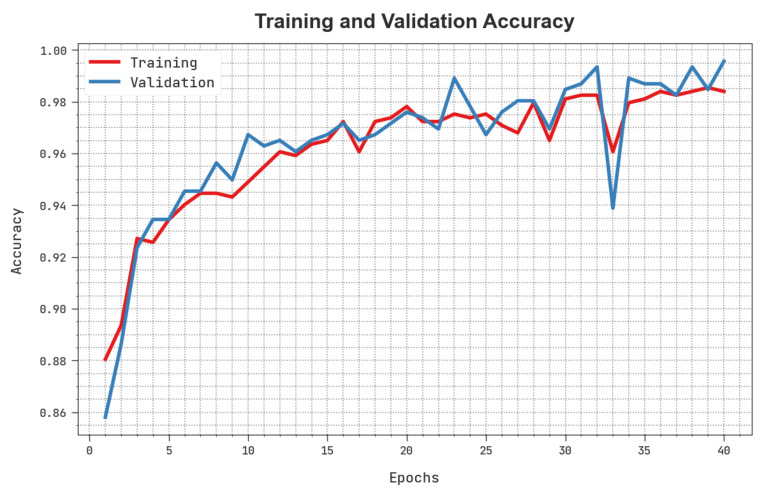
TACC and VACC analysis of HBODL-AOC approach.

The training accuracy (TACC) and validation accuracy (VACC) of the HBODL-AOC method is inspected on OC performance in Figure 7. The result implied that the HBODL-AOC technique had displayed improved performance with increased values of TACC and VACC. It is seen that the HBODL-AOC method has reached maximum TACC outcomes.

**Figure 8:**
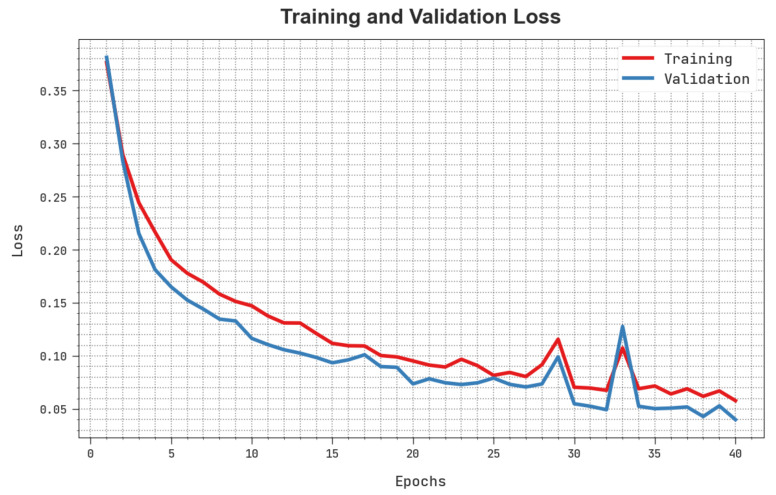
TLS and VLS analysis of HBODL-AOC approach.

The training loss (TLS) and validation loss (VLS) of the HBODL-AOC technique are tested on OC performance in Figure 8. The figure inferred that the HBODL-AOC approach had revealed better performance with the least values of TLS and VLS. It is noted that the HBODL-AOC technique has resulted in reduced VLS outcomes.

**Figure 9:**
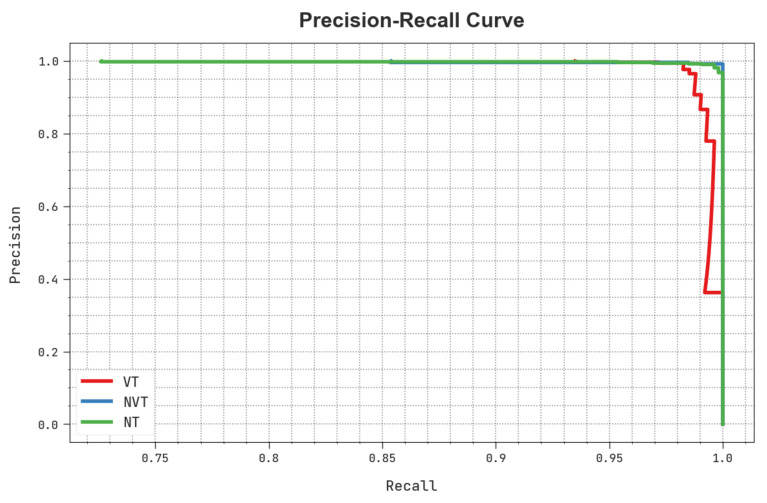
Precision-recall analysis of HBODL-AOC approach.

A clear precision-recall study of the HBODL-AOC technique under the test database is portrayed in Figure 9. The figure noted that the HBODL-AOC methodology has resulted in enhanced values of precision-recall values under all classes.

**Figure 10:**
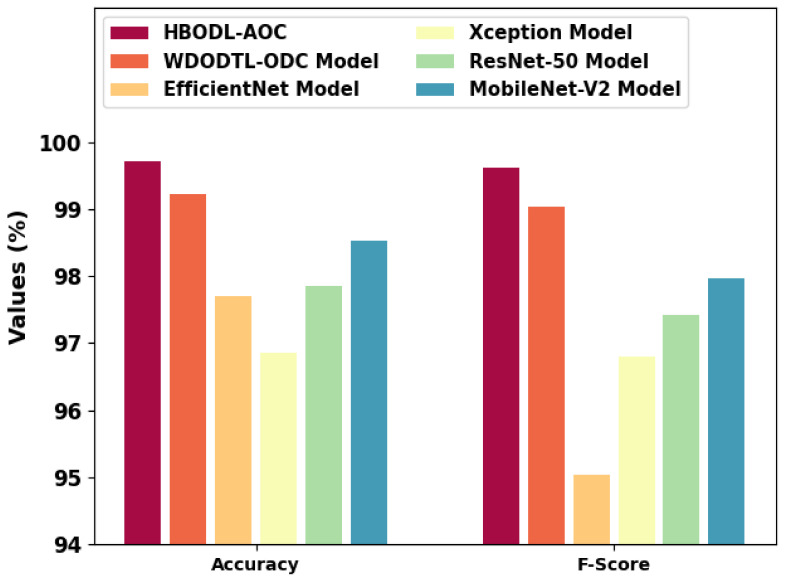
$Accu_{y}$ and $F_{score}$ analysis of the HBODL-AOC approach with other algorithms.

In Table 3, an extensive comparative study of the HBODL-AOC model with other DL models on OC classification is provided [24]. Figure 10 represents a comparative $accu_{y}$ and $F_{score}$ inspection of the HBODL-AOC method with other existing methods. The results show the HBODL-AOC model has attained higher values of $accu_{y}$ and $F_{score}$. Based on $accu_{y}$, the presented HBODL-AOC model has obtained improved $accu_{y}$ of 99.71%, while the wind-driven optimization with deep transfer learning enabled osteosarcoma detection and classification (WDODTL-ODC), EfficientNet, Xception, ResNet-50, and MobileNet-v2 models have reached reduced $accu_{y}$ of 99.22%, 97.70%, 96.85%, 97.85%, and 98.53% respectively.

Moreover, depends on $F_{score}$, the presented HBODL-AOC method has acquired improved $F_{score}$ of 99.62%, while the WDODTL-ODC, EfficientNet, Xception, ResNet-50, and MobileNet-v2 models have reached reduced $F_{score}$ of 99.04%, 95.03%, 96.80%, 97.42%, and 97.97% correspondingly.

**Figure 11:**
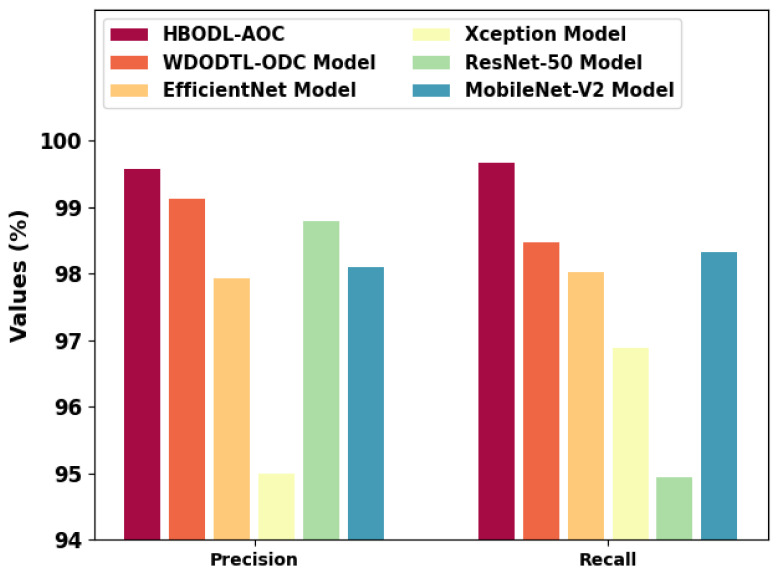
$Prec_{n}$ and $Reca_{l}$ analysis of the HBODL-AOC approach with other algorithms.

Figure 11 represents a comparative $prec_{n}$ and $reca_{l}$ analysis of the HBODL-AOC technique with other existing methods. The figure exhibited that the HBODL-AOC approach has attained higher values of $prec_{n}$ and $reca_{l}$. Based on $prec_{n}$, the presented HBODL-AOC model has obtained improved $prec_{n}$ of 99.57%, while the WDODTL-ODC, EfficientNet, Xception, ResNet-50, and MobileNet-v2 methodologies have reached reduced $prec_{n}$ of 99.13%, 97.94%, 95%, 98.80%, and 98.10% respectively. But based on $reca_{l}$, the presented HBODL-AOC model has obtained improved $reca_{l}$ of 99.68%, while the WDODTL-ODC, EfficientNet, Xception, ResNet-50, and MobileNet-v2 models have reached reduced $reca_{l}$ of 98.48%, 98.02%, 96.88%, 94.94%, and 98.33%, correspondingly. These results assured the better performance of the HBODL-AOC model over other DL models. The enhanced performance of the proposed model is due to the effective parameter selection of the MobileNet and ANFIS models.

## 5. Conclusions

In this article, we have introduced an Automated Osteosarcoma Classification model named the HBODL-AOC model. The goal of the presented HBODL-AOC technique is to identify the existence of osteosarcoma using medical images. In the presented HBODL-AOC technique, image pre-processing is initially performed by contrast enhancement technique. For feature extraction, the HBODL-AOC technique employed the MobileNet model with Adam optimizer for hyperparameter tuning. Finally, the HBO algorithm with the ANFIS model is applied for the osteosarcoma detection and classification process. To demonstrate the enhanced classification performance of the HBODL-AOC approach, a series of simulations were performed. The extensive simulation analysis portrayed the improved performance of the HBODL-AOC technique over existing DL models with maximum accuracy of 99.71%. In the future, the performance of the HBODL-AOC technique can be improved by hybrid DL classification models.

## Figures and Tables

**Table 1:** OC analysis of HBODL-AOC approach on 60:40 of TR/TS databases.

Labels	Accuracy	Precision	Recall	F-Score	AUC Score	MCC
Training Phase (60%)						
VT	98.54	97.88	96.86	97.37	98.03	96.36
NVT	99.42	98.80	98.80	98.80	99.21	98.42
NT	98.83	98.48	99.09	98.78	98.84	97.67
Average	98.93	98.39	98.25	98.32	98.69	97.48
Testing Phase (40%)						
VT	99.56	98.72	100.00	99.35	99.67	99.03
NVT	100.00	100.00	100.00	100.00	100.00	100.00
NT	99.56	100.00	99.04	99.52	99.52	99.12
Average	99.71	99.57	99.68	99.62	99.73	99.38

**Table 2:** OC analysis of HBODL-AOC approach under 70:30 of TR/TS databases.

Labels	Accuracy	Precision	Recall	F-Score	AUC Score	MCC
Training Phase (70%)						
VT	99.00	100.00	96.54	98.24	98.27	97.57
NVT	99.25	97.99	98.98	98.48	99.16	97.99
NT	99.00	98.15	99.73	98.93	99.05	98.00
Average	99.08	98.71	98.42	98.55	98.83	97.85
Testing Phase (30%)						
VT	99.71	100.00	99.12	99.56	99.56	99.34
NVT	99.42	98.48	98.48	98.48	99.06	98.13
NT	99.71	99.39	100.00	99.70	99.72	99.42
Average	99.61	99.29	99.20	99.25	99.45	98.96

**Table 3:** Comparative analysis of HBODL-AOC technique with other methods.

Methods	$Accu_{y}$	$Prec_{n}$	$Reca_{l}$	$F_{score}$
HBODL-AOC	99.71	99.57	99.68	99.62
WDODTL-ODC Model	99.22	99.13	98.48	99.04
EfficientNet Model	97.70	97.94	98.02	95.03
Xception Model	96.85	95.00	96.88	96.80
ResNet-50 Model	97.85	98.80	94.94	97.42
MobileNet-V2 Model	98.53	98.10	98.33	97.97

## Data Availability

Data sharing is not applicable to this article as no datasets were generated during the current study.
